# Knowledge, attitudes, and practices of Cameroonian physicians with regards to acute pain management in the emergency department: a multicenter cross-sectional study

**DOI:** 10.1186/s12873-019-0260-3

**Published:** 2019-08-08

**Authors:** Paul Owono Etoundi, Junette Arlette Metogo Mbengono, Ferdinand Ndom Ntock, Joel Noutakdie Tochie, Dominique Christelle Anaba Ndom, Francky Teddy Endomba Angong, Gérard Beyiha, Jacqueline Ze Minkande

**Affiliations:** 1 Department of Anesthesiology and Critical Care, Faculty of Medicine and Biomedical Sciences, University of Yaoundé I, Yaoundé, Cameroon; 2 Department of Anesthesiology and Critical Care, Yaounde Central Hospital, Yaoundé, Cameroon; 3 Department of Anesthesiology and Critical Care, Douala General Hospital, Douala, Cameroon; 4 Department of Internal Medicine and Specialties, Faculty of Medicine and Biomedical Sciences, University of Yaoundé 1, Yaoundé, Cameroon; 5 Department of Anesthesiology and Critical Care, Gynaeco-Obstetrics and Paediatric Hospital, Yaoundé, Cameroon

**Keywords:** Knowledge, Attitudes, Practices, Doctors, Pain, Emergency department, Cameroon

## Abstract

**Introduction:**

Pain is the most frequent presenting complaint in patients consulting or admitted to the emergency department (ED). Thus, its acute management is often done by physicians working in the ED. These clinicians are often general practitioners and not emergency medicine physicians in resource-poor settings. Hence, a mastery of pain management by these physicians may be important in relieving acute pain. We aimed to assess the knowledge, to determine the attitudes and practices of physicians in the management of pain in EDs of Cameroon.

**Methods:**

We carried out a prospective cross-sectional study over 4 months in the year 2018. We enrolled all consenting physicians who were neither emergency medicine doctors nor anesthesiologists working at the EDs of five tertiary hospitals of Cameroon. Using a validated and pretested structured questionnaire, data on the knowledge, attitudes, and practices of acute pain management at the ED by these clinicians were studied. We used an externally validated score to assess the knowledge as either poor, insufficient, moderate or good.

**Results:**

A total of 58 physicians were included; 18 interns or residents and 39 general practitioners. Their mean age was 28.6 ± 3 years and their average number of years of practice was 2.9 years. The level of knowledge was rated “poor” in 77.6% of physicians. Being a general practitioner was significantly associated with a poor level of knowledge (*p* = 0.02; OR = 5.1). We found a negative and significant correlation between knowledge and years of practice (*p* = 0.04; r2 = 0.06). More than three-quarter (82.8%) of participants used a pain scale to evaluate the severity of pain. The most used scale was the Visual Analog Scale (56.9%). The most frequently used analgesic was paracetamol (98.3%), although only 3.5% of physicians correctly knew its half-life, delay of onset of action and duration of action.

**Conclusion:**

These findings suggest that physicians in EDs of Cameroon have poor knowledge and suboptimal practices in pain management. General practice and a greater number of professional experience seemed to favour these attitudes. Overall, there is an urgent need for refresher courses in acute pain management for physicians working in these resource-limited EDs.

**Electronic supplementary material:**

The online version of this article (10.1186/s12873-019-0260-3) contains supplementary material, which is available to authorized users.

## Background

Acute pain is the most common reason for consultation in the emergency department and its reported to occur at a global prevalence of 20% [1, 2]. According to the International Association for the Study of Pain, pain is an unpleasant emotional experience in response to a real or potential tissue injury [3]. It is a multidimensional symptom involving sensory, emotional, cognitive and behavioral components [4]. Etiologies of pain are many and characterized by the causes of pain, its nature, type, location and severity [3].

The emergency department (ED) of a health facility is often the first unit to encounter patients consulting for diverse forms of acute pains. Health professionals working in the ED, therefore, play a key role in the first-line management of this pain. It is worth to mention that the initial management of acute pain can have an impact on the subsequent medical or surgical treatment of the patient. The level of knowledge on pain management at the ED in a study carried out in Italy [5] and Iran [6] showed poor knowledge amongst physicians working at the ED. Likewise, evidence from a recent national survey on emergency and essential surgical care (ESSC), a WHO tool, suggests that pain and emergency care management are inadequate and most hospitals lack pain relief guidelines in Cameroon [7]. Furthermore, unlike high-income countries where acute pain management at the ED is done by pain specialists such as emergency medicine physicians, anesthesiologists or board-certified emergency room general practitoners, in Cameroon, acute pain management is often done by general practitoners due to the paucity of pain management specialists. Hence, most Cameroonian EDs are run by general practioners whose knowledge, attitudes and practices towards acute pain management remain questionable till date. Hence, to improve the initial care of patients presenting with acute pain in the ED, we proposed this study to assess the level of knowledge, attitudes, and practices with regards to acute pain management in clinicians working in the EDs of major referral hospitals of Cameroon.

## Methods

### Study design and setting

This was a multicentre cross-sectional study with prospective collection of data between June 1, 2018, to September 30, 2018, in Cameroon. Cameroon is a sub-Saharan African country located precisely in central Africa within the Gulf of Guinea. The population of Cameroon is estimated between 22 and 24 million inhabitants [7, 8]. Here, health facilities are categorized into primary, secondary and tertiary hospitals depending on if they are sub-divisional/district, regional or general hospitals respectively [7]. Generally, almost all hospitals in Cameroon have an ED. However, the infrastructure for emergency and essential surgical care (ESSC) vary according to the category of the hospital [7]. Hence, tertiary hospitals have greater ESSC resources, compared to primary and secondary health facilities [7]. According to the ESSC, a WHO tool for situational analysis to assess ESSC, all categories of Cameroonian hospitals as well as their EDs have an inadequate oxygen supply, most lack a blood bank, physician specialists such as anesthesiologists and obstetrician/gynecologists are scant and majority lack a pain relief and emergency care management guidelines [7]. Also, Cameroon counts less than 10 emergency medicine physicians or board-certified emergency room general practitoners. Hence, most EDs are taken charged by general practitoners who take daily and night shifts.

### Study participants and sampling

The study population consisted of all consecutive consenting physicians working at the ED of five tertiary hospitals in Yaounde, the political city of Cameroon. Physicians were interviewed once without follow-up. The five hospitals were the Yaoundé Gyneco-Obstetrics and Pediatrics Hospital (HGOPY), the Yaoundé General Hospital (HGY), the Yaoundé National Emergency Center (CURY), the Yaoundé University Hospital Center (CHUY) and Yaoundé Central Hospital (HCY). We excluded emergency medicine and anesthesiologist physicians to reduce bias, given that by virtue of their profession, they were trained to manage acute pain compared to general practitoners and residents/interns of other specialties. Hence, given than most of the EDs in tertiary hospitals of Cameroon are ran by general practioners and residents or interns of other specialties, we sought to evaluate their knowledge, attitudes, and practices towards acute pain in a bit to assess their mastery of pain management at the ED.

### Definition of terms

Acute pain was defined as pain of fewer than 3 month’s duration. We defined nociceptive pain as a pain resulting from tissue lesions while neurogenic pain as pain due to a nerve lesion or compression.

### Data management and analysis

All participants had an interview-administration of a pre-tested 32-item questionnaire (see Additional file 1) to assess their knowledge, attitudes, and practices with regards to acute pain management in the five aforementioned EDs. The questionnaire was administered by the first four co-authors. Cut-off points for knowledge validated by Essi MJ et al. for KAP studies [9] and are represented in Table 1. Knowledge was scored out of 32 and ranked as in Table 1 [9]. On the other hand attitudes and practices were assessed both from the questionnaire and by self-assessment. Other data studied were socio-demographic variables. The data were coded, entered and analyzed using SPSS software version 20.0. The mean and standard deviation were used for the description of continuous variables. Categorical variables were described in terms of frequency and percentage. The association between categorical variables was measured by the Chi-square and Fisher tests. The association between two continuous variables was measured by the Pearson correlation test and materialized where appropriate by linear regression. When necessary, covariate adjustment by covariance analysis (ANCOVA) was used. The threshold of significance has been set at 5%.

**Table 1:** Correspondence between scores, percentages of correct answer and level of knowledge on acute pain management by physicians

Score	Percentage of correct answers	Level of knowledge
0–15	Less than 50%	Poor
16–20	Between 50 to 65%	Insufficient
21–27	Between 65 to 85%	Moderate
≥ 28	More than 85%	Correct

(developed by Essi MJ et al[10])

## Results

### Socio-demographic characteristics

A total of 75 physicians were interviewed of whom 58 consented to participate in the study. Hence, a response rate of 77.3%. The average age (standard deviation) of participants was 28.6 (3) years and the average number of years of experience (standard deviation) was 2.9 (2) years. The sex ratio was 1.14 in favor of men. Twenty-one (36.2%) of the participants practiced at the Yaoundé Central Hospital. More than half of the participants (67.2%) were general practitioners (Table 2).

**Table 2:** Socio-demographic characteristics

Variable	Frequency (*n* = 58)	Percentage (%)
Hospitals		
CHUY	7	12.1
CURY	11	19
HCY	21	36.2
HGY	7	12.1
HGOPY	12	20.7
Gender		
Female	27	46.6
Male	31	53.4
Occupation		
Resident/Intern	19	32.8
General practitioner	39	67.2
Number of years of professional experience		
Less than 5 years	47	81
More than 5 years	11	19

*CHUY*: Yaoundé University Hospital Center, *CURY* Yaoundé National Emergency Center, *HCY* Yaoundé Central Hospital, *HGY* Yaoundé General Hospital, *HGOPY* Yaoundé Gyneco-Obstetrics and Pediatric Hospital

### Knowledge of pain in emergencies

On average, the level of knowledge of the physicians was rated “poor”, with a mean score (standard deviation) of 11.8 (4.3) and ranged from 3 to 24. This corresponded to 45 (77.6%) participants having a poor level of knowledge, 10 (17.2%) with an insufficient level of knowledge, and 3 (5.2%) has a moderate level of knowledge. No “correct” level of knowledge was seen amongst the participants.

The highest percentage of the correct answer (93.1%) was obtained for the question regarding the types of analgesics used to treat pain. The lowest percentage (5.2%) was scored on the question regarding the number of types of pains. Residents and interns had significantly higher knowledge scores than general practitioners (*p* = 0.014). Being a general practitioner was significantly associated with poor knowledge (*P* = 0.02, OR = 5.1) compared to interns or residents. The number of years of professional experience was significantly and negatively correlated with the level of knowledge (*p* = 0.04 *r*^2^ = 0.06). The same was true for age (*p* = 0.02, r^2^ = 0.08). Hence, the older the age of the clinician and the higher his or her number of years of professional experience, the worse was his or her level of knowledge regarding the management of pain in the ED.

### Attitudes and practices of physicians regarding pain in the ED

Almost all participants (57/58) reported to maintain their calm when confronted with a patient in pain. For 38 (65.5%) participants, the pain was ranked among the first symptoms in patients consulting at the ED. Concerning participants’ practices towards pain, 38 (62.1%) administered an analgesic based on the patient’s complaint. Pain assessment was performed using the Visual Analogue Scale (VAS) in 56.9% by participants. The numerical scale was used by 13.8% of participants. Also, 10(17.2%) of the participants affirmed that their administration of analgesics was not based on any pain scale.

The decision to administer WHO group I analgesics was adequate in 42 (72.4%) of physicians. The administration of WHO group II and III analgesics was adequate in 40 (69%) of physicians. With regards to the most used analgesics, paracetamol was the most used analgesics by 57 (98.3%) physicians, followed by tramadol used by 44 (75.9%) physicians and diclofenac used by 22 (37.9%) physicians. Morphine was the least reported analgesic, used by only 5 (8.6%) physicians (Table 3). Paracetamol was the preferred analgesic used. The reason given by 26.3% of participants was due to its effectiveness in pain relief. Of the 57 physicians who reported using paracetamol in the ED, only two (3.5%) knew its correct half-life, delay of onset of action and duration of action. On the other hand, 6 (10.5%), 11(19.3%) and 15 (26.3%) physicians reported good answers concerning the half-life, the delay of onset of action and the duration of action of the drug, respectively.

**Table 3:** Attitudes and Practices towards acute pain

Variables	Frequency (*n* = 58)	Percentage (%)
Assessment of Pain		
Yes	48	82.8
No	10	17.2
Administration WHO group I analgesics		
Yes	42	72.4
No	16	27.6
Administration WHO group II analgesics		
Yes	40	69
No	18	31
Administration WHO group III analgesics		
Yes	40	69
No	18	31
Use of paracetamol		
Yes	57	98.3
No	01	1.7
Use of tramadol		
Yes	44	75.9
No	14	24.1
Use of diclofenac		
Yes	22	37.9
No	36	62.1
Use of Morphine		
Yes	05	8.6
No	53	91.4

*WHO* World Health Organization

## Discussion

This study is one of the first to assess the knowledge, to determine the attitudes and practices of physicians in the management of acute pain in the EDs of Cameroon. We found that the level of knowledge was “poor” and significantly associated with being a general practitioner. Furthermore, there was a negative and significant correlation between knowledge and years of practice. More than three-quarter of participants used a pain scale to evaluate the severity of pain. The most used scale was the Visual Analog Scale. The most frequently used analgesic was paracetamol, although only 3.5% of participants knew its exact half-life, delay of onset of action and duration of action.

The level of knowledge was averagely poor and lower than that found by Zanolin et al. [5] in Italy and by Kheshti et al. [6] in Iran using a similar cross-sectional survey as in the current study. This low level of knowledge on pain management in comparison with that found in the literature could be explained by the lack of refresher courses on acute pain management undertaken by clinicians working in Cameroonian EDs.

General practitioners had lower knowledge scores than residents and interns and their level of knowledge was negatively correlated to their number of years of professional experience. This may infer that the long years of clinical experience, Cameroonian general practitioners tend to take fewer refresher courses to maintain or upgrade their knowledge on pain management compared to physicians (residents and interns) undergoing a specialization program. This is in contrast with previous Lebanon cross-sectional study which showed inexperienced physicians to have a lower level of knowledge on pain management [10]. More contradicting results were obtained in an Iranian cross-sectional survey which observed a positive and significant correlation between the number of years professional experience and the level of knowledge on pain management (*p* = 0.002, *r* = 0.03) [6]. The discrepancy between our finding and the aforementioned studies [6, 10], may be explained by the absence of regular refresher courses organized in our resource-limited setting to continuously train and update physicians on pain management in the ED.

The most used scale to assess pain severity was the VAS (56.9%) probably explained by the simplicity of its clinical application. By contrast, in a similar-cross survey, Nasser et al. [10] found that the most used pain severity scale was the simple verbal scale also called the verbal rating scale (72.5%). The VAS was used only by 29% of physicians. The most used analgesic was paracetamol while the least used was morphine. This could be explained by physicians’ fear of the side effects of morphine especially respiratory distress which can be fatal in some patients.

The results from the present study should be interpreted within the context of its limitations. Firstly, its relatively small number of participants (*n* = 58), implying cautious generalization of findings to the entire Cameroon and other low-and middle-income countries. Nevertheless, we note a high response rate of 77.3% obtained and a multicenter study design conducted in five major referral hospitals of Cameroon, where the vast majority of physicians in the country work or train. With little or no similar prior studies, the current study is thus an invaluable contribution to the scarcity of data on the management of pain in the EDs of sub-Saharan Africa.

## Conclusion

This is one of the largest and first series to assess the knowledge, to determine the attitudes and practices of physicians in the management of acute pain in EDs of Cameroon and perhaps sub-Saharan Africa at large. This study shows the low level of physicians’ knowledge about acute pain management, as well as suboptimal practices, particularly in terms of acute pain management. General practice and a greater number of professional experience seemed to favour these attitudes. Overall, this study emphasizes an urgent need to organize regular refresher courses on acute pain management for clinicians working in EDs in resource-limited settings. Larger sample size and multinational studies are needed to provide more conclusive results.

## Additional file

Additional file 1: Questionnaire. (DOCX 28 kb)

## Data Availability

All data generated or analysed during this study are included in this published article.

## Abbreviations

CHUY: University Hospital Center of Yaoundé
CURY: Yaoundé National Emergency Center
ED: Emergency Department
HCY: Yaoundé Central Hospital
HGOPY: Obstetrics and Pediatric Gynecological Hospital of Yaoundé
HGY: General Hospital of Yaoundé
VAS: Visual Analog Scale
