# Supplementary material for: Knowledge, attitudes, and practices of Cameroonian physicians with regards to acute pain management in the emergency department: a multicenter cross-sectional study
Source: BMC Emerg Med. 2019 Aug 8;19:45. doi: 10.1186/s12873-019-0260-3 (PMC6688326; doi:10.1186/s12873-019-0260-3)
Supplement: Supplementary file 1 — Questionnaire. (DOCX 28 kb) [file 12873_2019_260_MOESM1_ESM.docx]

**Questionnaire**

**Knowledge, attitudes, and practices of Cameroonian physicians with regards to acute pain management in the emergency department: a multicenter cross-sectional study**

1. Identification
   1. Patient’s identification number : ____Age: ____ years: ____ Gender: _______
   2. Profession: Specialist? Yes / no: _________ Resident? Yes No: _________
   3. General practitioner yes / no: __________________
   4. Resident/Intern yes / no: ______________________
   5. Number of years of experience? ______________ years: _________

2. Knowledge

1. - Are there pain scales for adults? yes / no: ________________
2. What are the adult pain rating scales you know:
   1. Visual analogue scale yes / no: ______________________
   2. Numerical rating scale yes / no: _____________________
   3. Verbal rating scale yes / no: _________________________
   4. Caterorical scores yes / no: _________________________
   5. Comfort scale yes / no: _________________________
   6. McGill Pain Scale yes / no: _________________________
   7. Colour Analog Sale yes / no: _______________________
   8. Mankoski Pain Scale yes / no: ______________________
   9. Brief Pain Inventory yes / no: ______________________
3. - Are there pain scales for newborns and children who cannot talk? yes / no: ________
4. What are the pediatrics pain rating scales you know:
   1. Premature infant pain profile yes / no: ________________
   2. Colour Analog Sale yes / no: _______________________
   3. FLACC scale yes / no: _________________________
   4. Neonates pain rating Scale yes / no: ________________
   5. Face scale assessment tool yes / no: ________________
   6. Wong-Bank Faces Pain score yes / no: ______________
   7. Behavioral scale yes / no: _________________________
   8. PAT tool yes / no: _________________________
   9. Wong-Bank Faces Pain score yes / no: ______________
   10. CHEOPS Scale yes / no: _________________________
5. – According to the World Health Organization, how many levels of analgesics do you know ? (circle the right answer)
   1. 1
   2. 2
   3. 3
   4. All of the above
6. – According to your answer in the preceeding question, how many analgesics do you know in each level: ? (circle the right answer)
   1. 1: Paracetamol, morphine, non-steroidal anti-inflammatory drugs, nefopam
   2. 2: Tramadol, codeine, non-steroidal anti-inflammatory drugs
   3. 3: morphnie, morphine derivatives, tramadole, ketamine

g) How many types of pain are there? (Circle the right answer)

- 1. 1
  2. 2
  3. 3
  4. All of the above

h) Name the different types of pain you know. (Circle the right answer)

- 1. Nociceptive pain
  2. Neurogenic pain
  3. All of the above

i) Your assessment of pain in the emergency department is often based on which score or scale mentioned above? : ________________

j) Based on the answers provided in question ‘’i’’ above, what are the most prescribed analgesics drugs you used at the emergency department

- 1. Paracetamol
  2. non-steroidal anti-inflammatory drugs
  3. nefopam
  4. tramadol
  5. codeine
  6. specify if others: ________________

k) Can we make associations of analgesics for the management of pain? yes / no: _______

L) If yes, which levels can be associated with emergencies?

1. Level 1 and 2
2. Level 1 and 3
3. Level 2 and 3
4. Level 1, 2 and 3
5. Attitudes and Practices
6. After administering the analgesic, do you reasses the severity of pain to know if t=it is relief or not? yes / no: _______
7. What are the different severities of pain that you face at the emergency department?
   1. Mild
   2. Moderate
   3. Severe

iii. Do you perceive your patients are pain relieved after administering your analgesics? yes / no: _______

iv. At what rank does the pain in the symptoms most often found in the emergency department?

V. When do you administer an analgesic?

1. After a complete clinical examination? yes / no: ________________
2. After pain severity assessment? Y yes / no: ___________________
3. According to the type of pain? yes / no: _______________________
4. When do you administer level 1 analgesic? ___________________
   1. Mild pain
   2. Moderate pain
   3. Severe pain
   4. All the above
5. When do you administer stage 2 analgesics?
   1. Mild pain
   2. Moderate pain
   3. Severe pain
   4. All the above
6. When do you administer stage 3 analgesics?
   1. Mild pain
   2. Moderate pain
   3. Severe pain
   4. All the above

Vi. What are the analgesics you handle regularly in emergencies: _______________________

- 1. Fill in the blank box

| **Name** | **Half-life** | **Delayed onset of action** | **Duration of action** |
| --- | --- | --- | --- |
| Paracetamol |  |  |  |
| Diclofenac |  |  |  |
| Ketoprone |  |  |  |
| Piroxicam |  |  |  |
| Nefopam |  |  |  |
| Tramadol |  |  |  |
| Codeine |  |  |  |
| Ketamine |  |  |  |
| morphine |  |  |  |

Viii. Which level of analgesic do you prefer? ______________________________________

xi.Why ?_________________________________________________________

Ix. How do you evaluate pain management over time?

1. Pain severity scores
2. In relation to the onset of action of the analgesic?
3. In relation to the duration of action of the analgesic?
4. Patient’s verbal reponse of ‘’pan relief’’
5. All of the above
